# Prevalence of *Enterocytozoon bieneusi* and *Encephalitozoon* spp. in Swedish patients with suspected gastrointestinal parasite infection: a cross-sectional molecular study

**DOI:** 10.1007/s10096-026-05443-2

**Published:** 2026-02-25

**Authors:** Jessica Ögren, Anna J Henningsson, Silvia Botero-Kleiven, Andreas Matussek, Peter Wilhelmsson

**Affiliations:** 1 Department of Clinical Microbiology in Jönköping, Linköping University, Region Jönköping County, Jönköping, Sweden; 2 Department of Biomedical and Clinical Sciences, Division of Inflammation and Infection, Linköping University, Linköping, Sweden; 3 Department of Clinical Microbiology, Karolinska University Hospital, Stockholm, Sweden; 4 Department of Microbiology, Division of Laboratory Medicine, Oslo University Hospital, Oslo, Norway; 5 Department of Microbiology, Division of Laboratory Medicine, University of Oslo, Oslo, Norway

**Keywords:** Enterocytozoon bieneusi, *Encephalitozoon* spp., Microsporidia, Immunocompromised patients, Molecular diagnostics, Intestinal parasites

## Abstract

**Purpose:**

Microsporidia are opportunistic fungal parasites that may cause gastrointestinal disease in humans, mainly in immunocompromised individuals; yet contemporary prevalence data from Northern Europe are limited. The purpose of this study was to determine the prevalence of *Enterocytozoon bieneusi* and *Encephalitozoon* spp. in fecal samples from immunocompromised patients compared with reference patients without documented immunosuppression in Sweden.

**Methods:**

In this cross-sectional study, 1,238 fecal samples submitted for routine intestinal parasite diagnostics in 2023 at two Swedish microbiology laboratories were included. Samples originated from 619 immunocompromised patients and 619 age- and sex-matched reference patients. Real-time PCR was used to detect *E. bieneusi* and *Encephalitozoon* spp. All samples were additionally analyzed for *Entamoeba histolytica*, *Cryptosporidium* spp., and *Giardia intestinalis* to enable comparison with other intestinal pathogens.

**Results:**

*Enterocytozoon bieneusi* was detected significantly more often in immunocompromised patients than in reference patients (5.0% vs. 2.0%, *p* = 0.002). No cases of *Encephalitozoon* spp. were identified. The overall prevalence of *E. bieneusi* was similar between the two study sites (3.3% and 4.0%). No significant differences between groups were observed for *Cryptosporidium* spp., *G. intestinalis*, or *E. histolytica*.

**Conclusion:**

Immunocompromised patients in Sweden have a higher prevalence of *E. bieneusi* compared with reference patients. These findings provide updated prevalence data from a low-endemic setting and support the consideration of molecular diagnostics for microsporidia in selected patient groups.

## Introduction

Gastrointestinal illness is a significant global health burden, with bacteria, viruses, and parasites among the leading causes. Within this spectrum, microsporidia— a group of unicellular, spore-forming parasitic pathogens related to the kingdom Fungi have emerged as important opportunistic pathogens worldwide [1–3].

Opportunistic infections typically occur when host immunity is compromised, for example in HIV infection, during chemotherapy, autoimmune disease treatment, or following organ and bone marrow transplantation [4].

While usually self-limiting in immunocompetent individuals, enteric microsporidian infections in immunocompromised hosts may cause chronic diarrhea, malabsorption, and systemic disease [3, 5, 6]. Humans acquire infection mainly through ingestion of spores from contaminated food or water. To date, 17 species have been identified in humans [7], of which *Enterocytozoon bieneusi* and *Encephalitozoon* spp. (*E. cuniculi*, *E. hellem*, *E. intestinalis*) are the most common [3, 6]. *Enterocytozoon bieneusi* is the leading cause of intestinal microsporidiosis followed by *E. intestinalis* and *E. hellem* [8], while *E. cuniculi* is more associated with extraintestinal locations [7]. To date, only two outbreaks of foodborne microsporidiosis have been reported worldwide, both in Europe, including Sweden (2009) and Denmark (2020), highlighting the public health relevance of *E. bieneusi* [3, 9–11]. Reported global prevalence of *E. bieneusi* and *Encephalitozoon* spp. varies widely between studies, largely due to differences in geographic distribution, host populations, diagnostic methods, and sampling strategies [5, 6].

Diagnosis has traditionally relied on fecal microscopy, a labor-intensive method with limited sensitivity and poor species differentiation [12]. Although microsporidia are now classified as organism related to Fungi, their detection has remained part of the diagnostic framework for intestinal protozoa in most laboratories. Accurate species identification is essential for targeted treatment, epidemiological studies, and understanding the clinical relevance of different microsporidia [3, 7]. Molecular techniques have now largely replaced microscopy, providing higher sensitivity and supporting multiplex approaches for simultaneous detection of multiple enteric pathogens [13, 14]. However, optimal diagnostic algorithms and the clinical relevance of specific microsporidia remain subjects of debate.

In Sweden, data on human microsporidia infections are scarce; analysis for microsporidia is not part of routine testing algorithms for patients with gastrointestinal symptoms in Sweden, and studies largely predate molecular diagnostics, focusing primarily on HIV-infected patients [15–21]. As such, they may not accurately represent the current epidemiology of microsporidia in the country, highlighting the need for updated prevalence studies.

The present study aimed to determine the prevalence of *E. bieneusi* and *Encephalitozoon* spp. in fecal samples submitted for routine intestinal parasite testing at two separate Swedish university hospital laboratories. Using molecular methods, we provide current prevalence data to inform screening strategies and enhance the understanding of intestinal microsporidiosis, especially in immunocompromised patients.

To compare the prevalence of *E. bieneusi* and *Encephalitozoon* with intestinal protozoa in the different groups and between the sites, all samples were analyzed by real-time PCR for *Entamoeba histolytica*, *Cryptosporidium* spp. and *Giardia intestinalis* [22] in addition to being analyzed for *E. bieneusi* and *Encephalitozoon* spp.

## Materials and methods

### Sample collection and study groups

Fecal samples (*n* = 1,238) submitted for routine intestinal parasite diagnostics in 2023 were collected from two Swedish microbiology laboratories. Site one was the microbiology laboratory at Karolinska University Hospital, a tertiary care center serving approximately 2.3 million inhabitants in the Stockholm region, including large transplant, oncology, and haematology units. Site two was the microbiology laboratory at Ryhov County Hospital, Region Jönköping, a secondary care center serving approximately 340,000 inhabitants. Both laboratories function as complementary units within the Swedish National Reference Laboratory for Parasites.

At site one, requests for parasitological testing are submitted through separate diagnostic pathways for immunocompromised and immunocompetent patients. At site two, a unified diagnostic protocol is used, but clinicians are instructed to provide relevant clinical information, including immunosuppression status. At both sites, more extensive microscopic examination is routinely performed for immunocompromised patients to improve detection of uncommon parasites, but detection of microsporidia is not included and is only done upon a specific clinical request which is scarce.

Patients with documented immunosuppression were classified as immunocompromised group 1 (IC1, site one, *n* = 533) and immunocompromised group 2 (IC2, site two, *n* = 86). For each immunocompromised group, an age- and sex-matched reference group was constructed: reference group 1 (R1, *n* = 533) corresponding to IC1 and reference group 2 (R2, *n* = 86) corresponding to IC2. The reference groups consisted of patients without documented immunosuppression at the time of sample submission. Only anonymized data on age, sex, and immunosuppression status (if provided) were available for analysis.

### DNA extraction of fecal samples and real-time PCR

Fecal samples were stored in their original sample tubes at 8℃ for a maximum of four weeks, and then at -20℃ for up to two months prior to analysis. All extractions and real-time PCR analyses were performed at site two. Approximately 0.2 g of homogenized fecal material was suspended in 300 µl of 1 x phosphate-buffered saline (PBS, pH 7.4) and 20 µL proteinase K, then incubated at 56℃ for 20 min before being frozen at -80℃ for at least 1 h and up to 24 h. The samples were then incubated at 98℃ for 20 min, and centrifuged at 10,000 × *g* for 1 min. DNA from the supernatants was then extracted using the TANBead OptiPure Viral kit for the Maelstrom 4810 (TANBead, Taiwan) according to the manufacturer’s instructions.

*Enterocytozoon bieneusi* and *Encephalitozoon* spp.–specific PCR primers and detection probes were used, and amplification reactions were performed with Phocine herpesvirus (PhHV) as the internal control, as published by Verweij et al. [13]. Real-time PCR was performed using a CFX96 Touch Real-Time PCR Detection System (Bio-Rad Laboratories, Hercules, CA, USA) for 40 cycles.

Samples were classified as negative only if the internal control (PhHV) amplified successfully and all other positive and negative controls performed as expected. Positive samples were classified as positive if they showed a Ct value ≤ 37.

### Statistical analysis

Comparisons of parasite prevalence between groups were performed using chi-square test when expected cell counts were ≥ 5; Fisher’s exact test was used for analyses with small expected frequencies. Differences in age distributions were assessed with the Mann–Whitney U test. A *p*-value < 0.05 was considered statistically significant. All statistical analyses were conducted using IBM SPSS Statistics (IBM Corp., Armonk, NY, USA).

### Ethical approval

The study was approved by the Ethics Review Authority, Sweden (Dnr: 2021-06515-01).

## Results

### Study groups

A total of 1,238 fecal samples were analyzed: 1,066 from site one (IC1 and R1, *n* = 533 each) and 172 from site two (IC2 and R2, *n* = 86 each). Patient ages ranged from 18 to 90 years (median 43 at both sites), with an even sex distribution (site one: 51% male; site two: 49% male).

In IC1, underlying conditions included HIV (*n* = 50), inflammatory bowel disease (IBD) (*n* = 35), bone marrow transplantation (*n* = 15), solid organ transplantation (*n* = 7), hematologic malignancies (*n* = 3), and immunomodulating treatment (*n* = 38), while in 385 cases the type of immunosuppression was not stated. In IC2, the main groups were HIV (*n* = 32), IBD (*n* = 15), bone marrow transplantation (*n* = 18), solid organ transplantation (*n* = 9), and unspecified immunomodulating treatment (*n* = 12) (Table 1).

**Table 1:** *Enterocytozoon bieneusi* real-time PCR results by type of immunosuppression (as stated on the laboratory request form)

Type of immunosuppression	Patients positive / Total analyzed (%)	
	IC1	IC2
HIV	6/50 (12%)	3/32 (9.4%)
IBD	2/35 (5.7%)	1/15 (6.7%)
Solid organ transplant	2/15 (13.3%)	1/9 (11%)
Bone marrow transplant	1/7 (14%)	0/18 (0%)
Other (treatments, blood malignancy)	1/41 (0.25%)	0/12 (0%)
No information given	14/385 (3.9%)	0/0 (0%)
Total	**26/533 (4.9%)**	**5/86 (5.8%)**

*IC1* Immunocompromised patients from site one, Karolinska University laboratory; *IC2* Immunocompromised patients from site two, County Hospital Jönköping; *HIV* Human Immunodeficiency virus; *IBD* inflammatory bowel disease

### Detection of *Enterocytozoon bieneusi* and *Encephalitozoon* spp. using real-time PCR

No samples showed a difference between the two replicates.

In IC1 (*n* = 533), *E. bieneusi* was detected in 26 patients (4.9%) compared with nine patients (1.9%) in R1 (*n* = 533) (Table 2). In IC2 (*n* = 86), *E. bieneusi* occurred in five patients (5.8%) versus two patients (2.3%) in R2 (*n* = 86). At site one, *E. bieneusi* prevalence was significantly higher in IC than in R patients (*p* = 0.003; Chi-square test). When combining both sites, this difference remained significant (*p* = 0.002; Chi-square test), though site two alone showed no significant group difference due to limited sample size (*p* = 0.44; Fishers exact test). The overall prevalence of *E. bieneusi* was 3.3% (35/1,066) at site one and 4.0% (7/172) at site two, with no significant inter-site variation (*p* = 0.59; Chi-square test). No sex-related differences were observed (3.5% in men vs. 3.1% in women, *p* = 0.73; Chi-square test). However, *E. bieneusi*-positive IC patients were significantly younger (median 46 years) than positive R patients (median 65 years, *p* = 0.02: Mann-Whitney U test). Results stratified by type of immunosuppression are summarized in Table 1.

No cases of *Encephalitozoon* spp. were identified in any group.

### Detection of protozoa using real-time PCR

In IC1, *Cryptosporidium* spp. were found in 12 patients (2.3%) and *G. intestinalis* in 20 (3.8%). In R1, the corresponding prevalences were 2.1% (11/533) and 3.2% (17/533), respectively, along with two cases of *E. histolytica* (0.4%) (Table 2). In IC2, *Cryptosporidium* spp. and *G. intestinalis* were each detected in two patients (2.3%), and *E. histolytica* in one (1.2%). In R2, *Cryptosporidium* spp. were detected in three patients (3.5%) and *G. intestinalis* in one (1.2%), with no *E. histolytica* cases (Table 2).

**Table 2:** Real-time PCR results from fecal samples in the study groups for *Enterocytozoon bieneusi*, *Encephalitozoon* spp., *Cryptosporidium* spp., *Giardia intestinalis*, and *Entamoeba histolytica*

Microorganism	IC1 (%)	R1 (%)	IC2 (%)	R2 (%)
*E. bieneusi*	26/533 (4.9%)	9/533 (1.9%)	5/86 (5.8%)	2/86 (2.3%)
*Cryptosporidium* spp.	12/533 (2.3%)	11/533 (2.1%)	2/86 (2.3%)	3/86 (3.5%)
*G. intestinalis*	20/533 (3.8%)	17/533 (3.2%)	2/86 (2.3%)	1/86 (1.2%)
*E. histolytica*	0	2/533 (0.4%)	1/86 (1.2%)	0
*Encephalitozoon* spp.	0	0	0	0

*IC1* Immunocompromised patients from site one. Karolinska University laboratory R1, Reference group from site one IC2 Immunocompromised patients from site two. R2 Region Jönköping reference group from site two

There were no statistically significant differences between IC and R groups in the prevalence of *Cryptosporidium* spp., *G. intestinalis*, or *E. histolytica* (*p* = 0.53). Likewise, no significant inter-site variation was observed for these parasites (*p* = 0.79).

## Discussion

This study investigated the prevalence of *E. bieneusi* and *Encephalitozoon* spp. in immunocompromised and immunocompetent patients with suspected intestinal parasitic infections.

At both sites, *E. bieneusi* was detected more frequently among immunocompromised patients, supporting an association between immunosuppression and infection. A similar trend was seen across sites despite sample size differences, strengthening this observation. Interestingly, infected patients in the immunocompetent group were significantly older than infected patients in the immunosuppressed group, suggesting that advanced age may also increase susceptibility, which is in line with previous findings [23].

The overall prevalence of *E. bieneusi* was comparable between the two sites (3.3% vs. 4.0%), suggesting a relatively stable distribution despite differences in laboratory algorithms and geographical location. Notably, the prevalence observed in the reference groups in this study is higher than that reported in several other European settings, where prevalences of around 1% have been described in predominantly immunocompetent patient populations [24, 25] Furthermore, the prevalence reported here exceeds previous data from Sweden, where earlier studies based on microscopy identified microsporidia in only 0.2% of patients with enteritis (1/407) [17]. These differences are likely, at least in part, attributable to the increased sensitivity of molecular diagnostic methods compared with conventional microscopy. No cases of *Encephalitozoon* spp. were detected, consistent with previous reports identifying *E. bieneusi* as the dominant cause of human intestinal microsporidiosis [7]. Still, it cannot be excluded that the absence of *Encephalitozoon* spp. may partly reflect diagnostic limitations [11], underscoring the need for improved tools to detect less common species [26–28].

The highest infection rates of *E. bieneusi* were observed among transplant recipients and HIV-positive patients, consistent with earlier studies [3, 5]. However, it should be noted that this study was based on patients investigated for suspected parasitic disease rather than on a systematically screened population. Although viral load and CD4 + cell counts were not available, earlier studies have demonstrated strong associations between microsporidiosis and advanced immunosuppression [6, 29, 30]. Microsporidia are recognized as cosmopolitan pathogens, and intestinal microsporidiosis occurs in settings both endemic and non-endemic for parasites [31–33]. Nevertheless, exposure history may influence clinical suspicion. In Sweden, a substantial proportion of HIV-positive individuals are believed to have acquired their infection abroad [34], often in regions where parasitic infections are endemic. This may increase clinicians’ awareness and lower the threshold for parasitological testing in this group compared with patients with other forms of immunosuppression presenting with gastrointestinal symptoms. Such differences in testing practices may have contributed to selection bias and could partly explain the higher proportion of HIV-positive patients among those with detected *E. bieneusi*.

The lack of standardized travel and exposure data limits further interpretation, and systematic collection of such information would strengthen future prevalence estimates.

Interestingly, *E. bieneusi* was also detected in 6% of IBD patients (Table 1). Given Sweden’s high incidence of IBD [35, 36], and the use of immunomodulatory therapy, this finding is noteworthy. Whether microsporidia may influence IBD pathogenesis or represent opportunistic colonization remains unclear, but the overlap warrants further study, as suggested by previous reports [37–39].

Differences in how clinical data were recorded at the two sites highlight an important limitation. At site one, immunosuppression could be indicated without specifying the underlying condition, while at site two, detailed clinical data were available only when provided by the clinician. As a result, the type of immunosuppression was unknown for a large proportion of patients, reducing the ability to link infection risk to specific conditions. Nevertheless, sample distributions suggest that misclassification is unlikely to have affected the overall conclusions. These findings emphasize the need for standardized and detailed clinical information when submitting samples for parasitological testing [14]. The cross-sectional design of this study precludes conclusions regarding the timing of infection, causal relationships between immunosuppression and *E. bieneusi* infection, or associations between *E. bieneusi* detection and specific gastrointestinal symptoms. This study is also limited by the fact that samples were not analyzed by microscopy, nor was genotyping of the cases performed, which would allow epidemiological relationships or the risk of zoonotic transmission to be studied.

Importantly, *E. bieneusi* was the most common parasite detected in immunocompromised patients, yet it is not included in routine diagnostics in Sweden. This gap in clinical practice mirrors challenges also seen with *Cryptosporidium*, where nonspecific symptoms and lack of testing guidelines complicate diagnosis [40, 41]. The observed prevalence and previous reports in immunocompetent individuals [17] in addition to detection in animals and wastewater in the country [18, 20] and previously reported outbreaks of foodborne transmission [9, 10] highlight the public health importance of *E. bieneusi* and the need for rapid and accessible diagnostic methods.

In conclusion, this study demonstrates that immunocompromised patients in Sweden are at increased risk of *E. bieneusi* infection. Enhanced awareness, standardized diagnostic protocols, and more detailed clinical data would improve both patient management and epidemiological understanding. Further research should clarify the role of microsporidia in specific patient groups and guide evidence-based diagnostic strategies.

## Data Availability

The data supporting the conclusions of this article are included within the article. Raw data can be shared with researchers upon request.
